# JMCB Symposium 2019: The Legend of p53 vs. Cancer

**DOI:** 10.1093/jmcb/mjz078

**Published:** 2019-08-20

**Authors:** Ping Zhang, Suzanne P Christen, Gareth L Bond

**Affiliations:** 1 Ludwig Institute for Cancer Research, Nuffield Department of Medicine, University of Oxford, Oxford OX3 7DQ, UK; 2 The Simons Center for Systems Biology, Institute for Advanced Study, Princeton, NJ 08540, USA

The exciting symposium hosted by *Journal of Molecular Cell Biology* (JMCB), with the theme ‘The Legend of p53 vs. Cancer’, took place in Hangzhou, China on May 10–12, 2019. The symposium provided the opportunity for delegates in the fields of p53 biology, epigenetics, and cancer research to share scientific updates and to bridge the gap between basic science and translational research. The delegates also enjoyed celebrations of the 40th anniversary of the discovery of p53, as well as the 50th anniversary of the discovery of RNA polymerases.

## The discovery of p53

The symposium kick-off began with a welcome from Hua Lu from Tulane University. Hua introduced the discovery of the p53 gene, early insights into p53’s tumor-suppressive activities, and its recognition as the most frequently mutated gene in human cancer genomes. Jiarui Wu from Institute of Biochemistry and Cell Biology (IBCB), Chinese Academy of Sciences (CAS) used his opening remarks to express his appreciation to the organizers and encouraged participants to engage in active and open discussions. Carol Prives (Columbia University), one of the keynote speakers, shared her current research exploring the roles of p53 and its key negative regulator Mdm2 in regulation of lipid metabolism.

Following the introductory session, an open discussion brought together Guillermina Lozano (University of Texas MD Anderson Cancer Center), Carol Prives, Robert G. Roeder (The Rockefeller University), Wei Gu (Columbia University), and all participants to consider the future of p53 research, focusing on topics such as the biophysical, biochemical, and atomic details of p53 activation; targeting the p53 pathway in cancer therapy; and tissue- and cell-specific p53-associated activities.

## Gene regulation and epigenetics

This session was started by Wei Gu with an introduction for Robert G. Roeder who discovered RNA polymerases I, II, III 50 years ago. The speakers were high-level experts in this field, and a brief summary of their talks is provided below: Robert G. Roeder, a pioneer in eukaryotic transcription, presented a short summary of the discovery and function of the diverse components of the transcriptional machinery. Yi Zhang (Harvard University) identified a new genomic imprinting that plays important roles in X-chromosome in-activation, placenta development, and somatic cell nuclear transfer reprogramming. Guohong Li (Institute of Biophysics, CAS) presented the structure and functions of higher-order chromatin structures in gene regulation and epigenetic inheritance. Guo-Liang Xu (IBCB, CAS; Fudan University School of Medicine) described the importance of Tet-mediated oxidative de-methylation in embryonic development and cell reprogramming. Wei-Guo Zhu (Shenzhen University) described the diverse roles of SIRT7, a nucleus sirtuin, in regulating DNA damage repair and p53 pathway genes. David M. Gilbert (Florida State University) introduced technologies and methodologies for investigating epigenetic states and 3D chromatin architecture and described how *cis*-regulatory elements control the architecture of the genome and replication timing.

## The fourth decade of p53

p53, the ‘Guardian of the Genome’ and ‘Cellular Gatekeeper’, is accepted as being involved in a wide range of biological pathways. However, in what contexts p53 exerts its diverse functions, and how p53’s co-factors and post-translational modifications mediate them remains poorly understood. In the first part of this session, keynote speaker Wei Gu, as well as Xin Lu (University of Oxford), Ygal Haupt (Peter MacCallum Cancer Centre), and Jiandong Chen (Moffitt Cancer Center) shared their studies on the roles of p53’s post-translational modifications and co-factors in transcription regulation and tumor suppression.

Approximately 50% of cancer genomes have p53 mutations, and the vast majority of p53 mutations exert gain-of-function properties. In the second part of this session, Giannino Del Sal (University of Trieste & IFOM), Wenwei Hu (Rutgers University), Gareth L. Bond (University of Oxford), Jinrong Peng (Zhejiang University), Zhi-Xiong Xiao (Sichuan University), and Peng Jiang (Tsinghua University) spoke about how oncogenic mutant p53 and p53 isoforms affect tumorigenesis, and how common inherited mutations in p53 pathway genes interact with somatic p53 mutations to affect cancer risk and progression.

## The p53 network

To maintain genome integrity, p53 controls a wide and context-specific signaling network that is involved in hundreds of genes regulating cell cycle arrest, senescence, and apoptosis, as well as stem cell pluripotency, cellular plasticity, metabolic pathways, and ferroptosis. Keynote speaker Guillermina Lozano shared her research exploring mutant p53 activities in a somatic model of breast cancer, looking at the microenvironment in tumor development and progression using mouse genetics. Yanping Zhang (University of North Carolina at Chapel Hill) spoke about the importance of the p53–MDM2/MDMX interplay in development and radiation sensitivity. Hua Lu spoke about the roles of the ribosomal stress–MDM2–p53 pathway in cancer cell proliferation and tumorigenesis and mechanisms underlying these phenotypes. Xin-Hua Feng (Zhejiang University) discussed the effects of loss-of-function mutations and ALK-mediated phosphorylation of the SMAD4 gene on TGF-β resistance. Zhaohui Feng (Rutgers University) talked about how the p53 target gene Parkin inhibits glycolysis and cancer metastasis through ubiquitin-mediated HIF-1α degradation. Hai Jiang (IBCB, CAS) presented a novel strategy to define hotspot p53 mutations by integrating both the original mutation counts and their relative mutational difficulty in cancer genomes. Bin-Bing Zhou (Shanghai Children’s Medical Center) described the role of p53 mutations in inducing resistance to chemotherapies for acute lymphoblastic leukemia. Xiang Zhou (Fudan University) described the interplay between ubiquitin ligase TRIM71 and mutant p53 in ovarian cancer.

The ability of p53 to induce permanent cell cycle arrest and death is well known, given that these functions are commonly inactivated through mutation in many cancer types. However, recent data in model systems suggest that the pro-survival roles of wild-type p53 could also be involved in tumorigenesis, tissue homeostasis, and metabolism. Yang Xu (University of California, San Diego) presented an oncogenic role of wild-type p53 in hepatocarcinoma cells, where p53 promotes a cancer metabolic switch by inducing PUMA-mediated disruption of oxidative phosphorylation. Zhi-Min Yuan (Harvard School of Public Health) showed that p53 enhances ferroptosis by impeding Slc7a11 expression, which stimulates hepatocyte proliferation and liver regeneration.

## Bringing p53 biology into the clinic

Oncogene MDM2, the key p53 negative regulator, is amplified and/or overexpressed in a variety of cancers, and its amplification has been found to result in increased cancer susceptibility, tumor growth and metastasis, as well as a weaker, p53-mediated, DNA damage response and resistance to therapy. Thus, MDM2 has become a promising target for treatment in combination with DNA-damaging therapies, and various MDM2 inhibitors are currently being developed and tested (in clinical trials). Shaomeng Wang (University of Michigan) described MDM2 degraders that can achieve complete tumor repression and greatly improve survival of animals in leukemia models. Ruiwen Zhang (University of Houston) presented his research on developing dual inhibitors for inflammatory factor NFAT1 and MDM2 for cancer prevention and treatment. Douglas Fang (Ascentage Pharma) shared data on a promising combination cancer therapy with MDM2 antagonist APG-115 and immune checkpoint blockade. Xin-Yuan Fu (Sichuan University) introduced discoveries about the STAT gene family and the druggable JAK–STAT pathway, and Weikang Tao (Jiangsu Hengrui Medicine) provided an overall summary of the company’s drug development pipeline, focusing on unmet medical need.

The extreme abundance of p53 driver mutations in cancer has motivated the development of small molecules, antibodies, and siRNAs that aim to reactivate mutant p53 in cancers. Kanaga Sabapathy (National Cancer Centre Singapore) shared his research on targeting individual mutant p53 for cancer prevention and treatment by using mutation-specific antibodies and siRNAs. Min Lu (Shanghai Institute of Hematology) discovered a small molecule that can modulate a batch of structural p53 mutants to restore wild-type-like transcription and tumor-suppressive activities in cancer.

At last, Guillermina Lozano gave the concluding remarks pointing out that p53 is still an enigma with emerging themes to understand or explain. We look forward to hearing more exciting progress in the near future.

